# New record of *Microtusmystacinus* in eastern Kazakhstan: phylogeographical considerations

**DOI:** 10.3897/zookeys.781.25359

**Published:** 2018-08-13

**Authors:** Tereza Holicová, František Sedláček, Anna Mácová, Jakub Vlček, Jan Robovský

**Affiliations:** 1 Department of Zoology, Faculty of Science, University of South Bohemia, České Budějovice, Czech Republic University of South Bohemia České Budějovice Czech Republic; 2 Department of Parasitology, Faculty of Science, University of South Bohemia, České Budějovice, Czech Republic University of South Bohemia České Budějovice Czech Republic

**Keywords:** *
Microtus mystacinus
*, Kazakhstan

## Abstract

The Eastern European vole (*Microtusmystacinus*) is an arvicoline rodent distributed across northern and eastern Europe, the Balkans, Turkey, Armenia, NW and N Iran, Russia as far east as the Tobol River in W Siberia, and W and N Kazakhstan. We present a novel records from eastern Kazakhstan (the village of Dzhambul – 49°14'21.3"N, 86°18'29.9"E and the village of Sekisovka – 50°21'9.18"N, 82°35'46.5"E) based on mtDNA and we discuss implications of this findings on biogeography of eastern Kazakhstan populations. Marine Isotope Stage 11 is considered an important period for the diversification of the *arvalis* species group. In the context of our study, it is important to analyse genetically discontinuous Siberian populations, and the current distribution of *Microtusmystacinus* in new localities in eastern Kazakhstan.

## Introduction

The Eastern European vole, *Microtusmystacinus* De Filippi, 1865, is an arvicoline rodent with an unsettled nomenclature. It has been named most commonly as *M. subarvalis* Meyer, Orlov & Skholl, 1972, *Microtusepiroticus* Ondrias, 1966, *Microtusrossiaemeridionalis* Ognev, 1924, and *Microtuslevis* Miller, 1908 (e.g., Musser and Carleton 2005; Kryštufek and Vohralík 2005). We adhere to the name *Microtusmystacinus*, following the detailed study by Mahmoudi et al. (2017) and the review of Kryštufek (2017). Despite its nomenclature instability, there is a consensus about its phylogenetic affinities: this species has been traditionally attributed to the *arvalis* species group in the subgenus Microtus s. str. (Musser and Carleton 2005). This view has been strongly supported by chromosomal and genetic evidence (e.g., Mazurok et al. 2001, Jaarola et al. 2004, Mahmoudi et al. 2017). According to new studies, it is related to the following species: *M. ilaeus* Thomas, 1912 (syn. *M.kirgisorum* Ognev, 1950), *M.transcaspicus* Satunin, 1905, *Microtuskermanensis* Roguin, 1988, *Microtusarvalis* (Pallas, 1778), and *M.obscurus* (Eversmann, 1841) (e.g., Golenishchev et al. 2000; Jaarola et al. 2004; Kryštufek and Vohralík 2005; Mahmoudi et al. 2017), but it is the closest relative of *Microtusarvalis* and *M.obscurus* based on available DNA data (cyt *b*; Mahmoudi et al. 2017).

In general, *Microtusmystacinus* represents one of the best cases of a cryptic species in arvicolines, because it was primarily recognized by chromosomal number (*Microtusmystacinus*: 2n = 54; *Microtusarvalis*: 2n = 46) (Meyer et al. 1969; Mazurok et al. 2001; Pavlova and Tchabovsky 2011). It is now generally considered a valid species of the genus *Microtus* based on hybridisation data, and chromosomal and genetic differences (for reviews see Kryštufek and Vohralík 2005 and Musser and Carleton 2005). Several authors have attempted to distinguish *Microtusmystacinus* from the common vole (*Microtusarvalis*), the Altai vole (*M.obscurus*), and the Middle Eastern vole (*M.transcaspicus*) based on morphological data (Král et al. 1981; Zagorodnyuk 1991a, b; Masing 1999; Hotzi et al. 2008; Markova et al 2009, 2012; Markov et al. 2012; Ghorbani et al. 2015). Although some diagnostic characters have been proposed (e.g., qualitative and quantitative cranial and dental morphology) and multivariate morphometric approaches have been applied (e.g., Markov et al. 2012; Markova et al. 2012), these approaches have been lacking in diagnostic power (Kryštufek and Vohralík 2005; Markov et al. 2012), except for characters proposed by Kryštufek and Vohralík (2005).

The distribution and habitat preferences of the Eastern European vole are relatively well known due to the intensive attention devoted to it (see Kryštufek and Vohralík 2005; Musser and Carleton 2005; Shenbrot and Krasnov 2005; Kryštufek 2017, and references therein). It prefers to live in places with high and dense herbaceous or grassy vegetation, hedgerows, and stands of reeds and it avoids short-grass meadows and dry areas (Kryštufek and Vohralík 2005; Aulagnier et al. 2009; Kryštufek 2017). The distribution range of the Eastern European vole, to date, extends from southern Finland, the Baltic eastwards to western Siberia with patches in the southern Urals, the Novosibirsk suburbs to the southwest margin of Lake Baikal and Buryatia, the southern Caucasus, northern Iran to Turkey, connecting to Greece and the majority of the Balkan Peninsula to Ukraine (Baskevich 1996; Gileva et al. 1996; Yakimenko and Kryukov 1997; Musser and Carleton 2005; Shenbrot and Krasnov 2005; Pavlova and Tchabovsky 2011; Ghorbani et al. 2015; Baskevich et al. 2016; Kryštufek 2017; Moroldoev et al. 2017).

Populations occupying the Artic Svalbard Archipelago (Fredga et al. 1990; recently extinct according to Aulagnier et al. 2009), Jan Mayen Island in the N Atlantic (Kryštufek 2017), Olkhon Island in Lake Baikal (Pavlova and Tchabovsky 2011; Kryštufek 2017) and Far Eastern Russia (Khabarovsk Krai, near Sovetskaya Gavan City, see Kartavtseva et al. 2012; Tiunov et al. 2013) are probably introduced. *Microtusmystacinus*, *Microtusarvalis*, and *M.obscurus* broadly overlap in distribution and occur sympatrically in a few regions (e.g., Meyer et al. 1996; Musser and Carleton 2005; Shenbrot and Krasnov 2005 see also Tougard et al. 2013).

When considering the distribution of *Microtusmystacinus* within Kazakhstan, there are records from the western or north-western parts. The easternmost record is from the Karabalyk district (Kovalskaya 1994; Meyer et al. 1996). Here, we report an additional record of *Microtusmystacinus* from eastern Kazakhstan and comment on it from a phylogeographic point of view.

## Materials and methods

A survey of small mammals conducted in eastern Kazakhstan provided the surprising discovery of three specimens of *Microtusmystacinus*, that are characterized here based on molecular methods. The first sample (Kazakhstan 1) was collected in July 2006 on pasture land near the village of Dzhambul (GPS coordinates: 49°14'21.3"N, 86°18'29.9"E) by FS and two more specimens (Kazakhstan 2, 3) were collected in September 2017 near a pond not far from the village Sekisovka (GPS coordinates: 50°21'9.18"N, 82°35'46.5"E) by AM and JV.

DNA extraction was carried out using the Genomic DNA Mini Kit – tissue (Geneaid, New Taipei, Taiwan). We amplified the mitochondrial gene cytochrome *b* (cyt *b* hereinafter) using universal primers L14724, L15162, H15149 and H15915 (Irwin et al. 1991). Amplification conditions for cyt *b* consisted of 37 thermal cycles, an initial denaturation step at 94 °C for 3 min, denaturation at 94 °C for 30 seconds, annealing at 50 °C for 1 min, extension at 72 °C for 1.5 min and final extension at 72 °C for 10 min. Sequences were obtained using the Sanger sequencing (Sanger et al. 1977) services at laboratory SEQme s.r.o. (Dobříš, Czech Republic).

We obtained 1137 base pairs long sequences that satisfied the quality of base pairs (GenBank access number LT970847-LT970849). These were compared using available sequences from GenBank, specifically with 250 specimens that comprise all available sequences of *Microtusmystacinus* (under names *Microtuslevis, M. rossiameridionalis* and *Microtusmystacinus*), and representative sequences of particular clades in *Microtusarvalis* and *M.obscurus* associated with previous studies (Baker et al. 1996a, b; Haynes et al. 2003; Fink et al. 2004; Jaarola et al. 2004; Triant and DeWoody 2007; Bužan et al. 2010; Thanou et al. 2012; Tougard et al. 2013; Stojak et al. 2016; Mahmoudi et al. 2017). Several more sequences (*M. kirgisorum*, accession number AY513809, AY513810; *M. socialis*, accession number AY513830, AY513831; and *M.transcaspicus*, accession number KX581067-KX581075) were downloaded from GenBank as potentially outgroups. The obtained sequences were aligned using the ClustalW algorithm implemented in GENEIOUS v.10.0.5 (Kearse et al. 2012). We employed a likelihood (ML) and Bayesian inference method (BI) for phylogenetic analyses. Likelihood phylogenetic analyses were conducted using the PhyML plugin for GENEIOUS. Final Bayesian phylogenetic analyses were conducted in BEAST 2.4.5.0 (Drummond et al. 2012), where phylogenetic relationships were reconstructed under the Yule speciation process (Steel and McKenzie 2001) with the GTR model of evolution detected in JModelTest 2.1.7 (Nylander 2004) under the Akaike Information Criterion (AIC). The nucleotide data were run for 30 000 000 generations with a sampling frequency of every 1000^th^ generation; with final burn-in set at 20%. Time estimations were also computed in BEAST2 (Drummond et al. 2012) for the topology detected by the Bayesian phylogenetic analysis. We adopted one fossil calibration point (0.475±0.025 Mya for the origin of *Microtusarvalis*: Miesenheim I; Tougard et al. 2013) to estimate divergence time in studied taxa and to compare estimations with Mahmoudi et al. (2017) (which are based on the following proposed molecular clock rate, 3.27×10^-7^ mutations/site/year for *Microtusarvalis*; Martínková et al. 2013). The split time with 95% highest posterior density was applied to a relaxed-clock model assuming a constant population size. The convergence and stability of estimated parameters was checked using TRACER 1.6 (Rambaut et al. 2017) and the maximum clade credibility trees were obtained with TREEANNOTATOR 2.4.5.0, and visualized in FIGTREE 1.4.3 (Rambaut 2009).

Some analyses were applied for *Microtusmystacinus* only. Specifically, haplotype characteristics were identified using DnaSP version 5.0 (Rozas et al. 2003) and the degree of diversification was estimated based on average pairwise distances using the Kimura two-parameters model of substitutions in MEGA5 (Tamura et al. 2011). The detailed haplotype network was conducted in POP ART 1.7 using the median-joining method (Bandelt et al. 1999).

## Results and discussion

The obtained sequences of 1137 base pairs from three specimens exhibited close relationships with available cyt *b* sequences of *Microtusmystacinus*, in all comparisons. Specifically, they were nested inside this species, so our study identified this species in eastern Kazakhstan (see also below). All sequences of *Microtusmystacinus* form a sister group to the *Microtusobscurus* + *Microtusarvalis*, in accordance with previous comprehensive studies (e.g., Haynes et al. 2003; Fink et al. 2004; Jaarola et al. 2004; Triant and DeWoody 2007; Tougard et al. 2013; Stojak et al. 2015, 2016; Mahmoudi et al. 2017).

**Figure 1.**
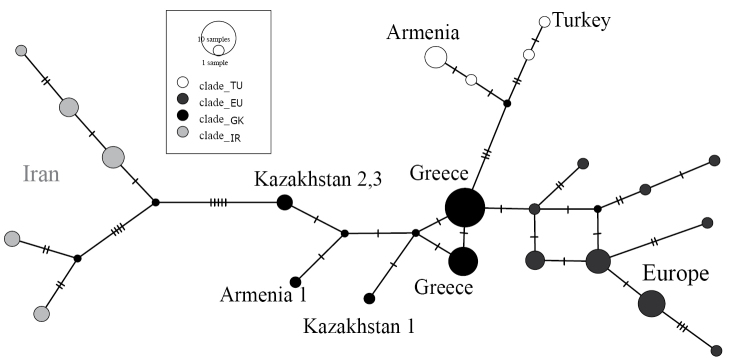
Median Joining Network based on the cyt *b* sequences of *Microtusmystacinus*.

Considering the intraspecific structure in *Microtusmystacinus*, we can distinguish two deep lineages (Iran, abbreviated as IR) and the rest of populations mostly from Europe, additionally divided into several sub-lineages (TU, EU, GK), concordantly in ML and BI phylogenetic trees and the haplotype network (see Figure 1). This structure, specifically groups IR, TU, and EU, were identified firstly by Mahmoudi et al. (2017). TU lineage consists of Turkish and Armenian samples (without specimen Armenia 1), EU lineage of samples from the majority of Europe, mainly from Ukraine and Romania except for specimens from Greece, which comprise GK lineage, as well as samples from eastern Kazakhstan and the specimen 1 from Armenia. This pattern indicates a complex diversification of *Microtusmystacinus* across its former and current distribution.

In general, *Microtusmystacinus* exhibited rather low intraspecific cyt *b* distances (except for the Iranian subset) and the obtained interspecific cyt *b* distances (see Table 1) are very similar to the values published in other studies (*Microtusarvalis* × *mystacinus*: Jaarola et al. (2004): 6–8%; Mahmoudi et al. (2017): 6–7%). As the intraspecific divergence for *Microtusmystacinus* and its cryptic diversity was intensively discussed by Mahmoudi et al. (2017), we would like to note only that the genetic distances cannot be presented as an absolute criterion for deciding whether two operational taxonomic units are distinct species (for detail see Groves et al. 2017), and in the case of species within the *arvalis*-group, some currently recognized species with rather low genetic distances exhibit infertile hybrids or hybrids with a reduced fertility (Meyer et al. 1985; Golenishchev et al. 2000; Jaarola et al. 2004).

**Table 1.** The K2P Inter – and intra-species average estimates of K2 genetic distance for cyt *b* in recognized lineages of *Microtusmystacinus* (TU – Turkey, Armenia; EU – Europe; GK – Greece, Kazakhstan; IR – Iran).

	1.	2.	3.	4.	5.	6.	7.	8.	9.	10.
1. TU	0.007									
2. EU	0.025	0.007								
3. GR	0.021	0.016	0.006							
4. Armenia_1	0.024	0.019	×	×						
5. Greece	0.016	0.011	×	0.009	0.001					
6. Kazakhstan	**0.023**	**0.018**	×	**0.007**	**0.008**	**0.006**				
7. IR	0.035	0.044	0.031	0.031	0.034	**0.028**	0.013			
8. *M.obscurus*	0.067	0.066	0.065	0.062	0.066	0.059	0.068	0.028		
9. *Microtusarvalis*	0.067	0.057	0.065	0.062	0.066	0.063	0.067	0.059	0.003	
10. *M.transcaspicus*	0.075	0.079	0.071	0.069	0.072	0.065	0.068	0.067	0.084	0.004

The estimated clade divergence times varied substantially according to the calibration used (see Table 2). In summary, our estimations are more similar with other estimates based on fossil calibration points (albeit slightly higher) than with estimations based on mutation rates (see Table 2). Focusing on the most studied species, *Microtusarvalis*, we estimate its time to the most recent common ancestor (TMRCA) as 0.490 Mya, Tougard et al. (2008) 0.472 Mya and Tougard et al. (2013) 0.446 Mya, Stojak et al. (2015, 2016) 0.064-0.067 Myr and Mahmoudi et al. (2017) 0.146. Our estimation is similar to Tougard et al. (2008, 2013) as a logical result of the utilization of the same fossil calibration point, but all other specified estimations are much lower and associated with the same mutation rate (3.27 x10^-7^ substitutions/site/year) proposed by Martínková et al. (2013) specifically for *Microtusarvalis* based on a recent geological event. It is not easy to judge which values are realistic, but our estimates seem to be compatible with other phylogenetic studies (e.g., Mazurok et al. 2001; Bannikova et al. 2010) and the fossil record (e.g., Cuenca-Bescós et al. 2001; Markova et al. 2012). Based on this compatibility, we adhere to the values of our estimations. In any case, it would be worth to compare different calibrations methods under different calibrations points and proposed mutations rates in future (e.g., methods of Baker et al. 1996a; Jaarola and Searle 2002), and also to consider the potential biases of the fossil record (e.g., incomplete nature, process of geological dating, reliability of species identification; cf. Ho 2007).

**Table 2.** Time to the most recent common ancestor (TMRCA and 95% HPD lower/upper limit – in million years) with BEAST2 for particular *Microtus* species (T – *M.transcaspicus*, M – *Microtusmystacinus*. O – *M.obscurus*. A – *Microtusarvalis*) and recognized lineages of *Microtuslevis* (TU – Turkey, Armenia; EU – Europe; GK – Greece, Kazakhstan; IR – Iran).

**Nodes**	**Analysis 1 – fossil calibrations**		**Mahmoudi et al. 2017**	**Tougard et al. 2013**
	TMRCA	95% HPD	TMRCA (95%HPD)	TMRCA (95%HPD)
a. T+M+O+A	1.102	0.77–1.28	0.238 (0.16–0.35)	–
b. M+O+A	0.797	0.60–1.05	0.217 (0.15–0.31)	0.531 (0.42–0.67)
c. O+A	0.616	0.51–0.78	0.184 (0.12–0.26)	0.478 (0.40–0.56)
d. T	0.537	0.32–0.57	0.040 (0.01–0.08)	-
e. O	0.410	0.27–0.58	0.119 (0.07–0.18)	0.173 (0.10–0.29)
f. A	0.490	0.48–0.54	0.146 (0.10–0.21)	0.446 (0.39–0.49)
g. IR+ EU+GK+TU	0.575	0.04–0.77	0.147 (0.09–0.22)	0.033 (0.00–0.08)
h. EU+GK+TU	0.408	0.28–0.57	0.092 (0.05–0.14)	–
i. EU+GK	0.332	0.23–0.47	–	–
j. TU	0.235	0.10–0.40	0.022 (0.01–0.04)	–
k. EU	0.219	0.14–0.32	0.075 (0.05–0.11)	–
l. GK	0.280	0.19–0.40	–	–
m. IR	0.390	0.24–0.47	0.117 (0.06–0.18)	–

Evolution and diversification of arvicoline rodents, including the *arvalis*-group, has been closely related to Quaternary climatic oscillations and the associated abiotic and biotic environmental factors (e.g., Horáček and Ložek 1988; Horáček 1990; Chaline et al. 1999; Stojak et al. 2016; Tougard 2017 and references therein). For the *arvalis*-group, interglacial periods are considered to be periods of species expansions and glacials as periods of retractions with potential survival of particular species in refugia (e.g., Golenishchev et al. 2000; Tougard et al. 2008; Stojak et al. 2015; Stojak et al. 2016). Golenishchev et al. (2000) considered one of the ancient alpine glaciations as responsible for disrupting the geographic range of *Microtusarvalis* and *M.obscurus*, whereas Tougard et al. (2008) considered interglacials as the agents of speciation. Based on our time estimations, the diversification of *Microtusmystacinus* + (*Microtusarvalis* + *M.obscurus*) group has happened within the last 0.79 Mya, thus comprising several interglacial and glacial periods (Gates 1993; Sirocko et al. 2007; Mahmoudi et al. 2017).

**Figure 2.**
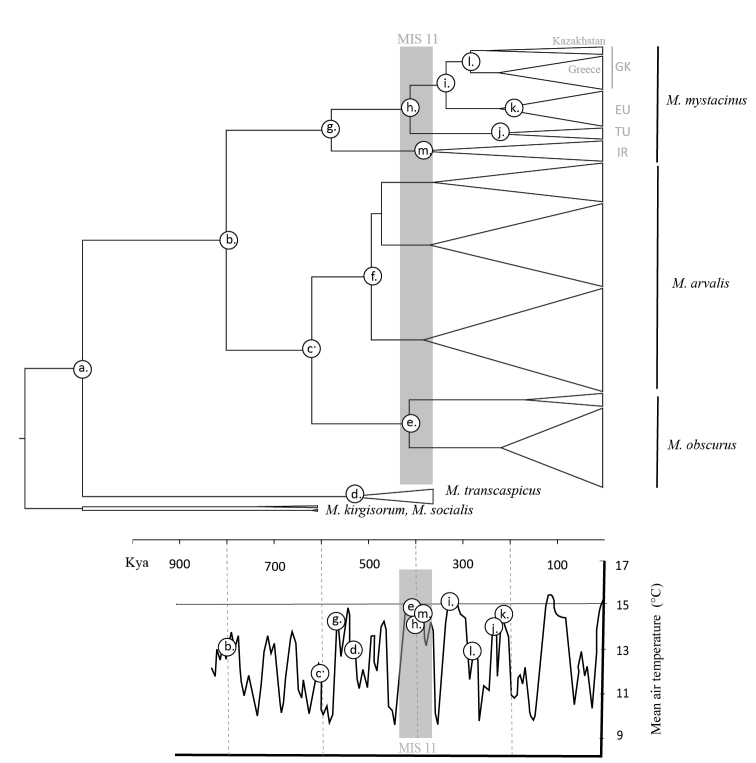
Time of the most recent common ancestor (TMRCA) for *Microtus* species and lineages of *Microtusmystacinus* using fossil calibrations. Nodes are plotted on a mean air temperature curve in last 800 thousand years (based on Gates 1993). See Table 2 for time estimates.

In our data, we observed synchronous, deep intraspecific divergences in all three species around 0.49–0.41 Mya (see Figure 2; in *M.mystacinus* we operated with separate timelines for the Iranian lineage (IR) and the remainder (sub-lineages TU, EU, GK) because the Iranian populations are divergent from the others; pairwise distance shows significant variation, see Table 1). This interval corresponds to the Holstein interglacial period (considering the stratigraphy of Western Europe) that is considered to be equivalent to Marine Isotope Stage (MIS) 11 (Sirocko et al. 2007; see Figure 2). The influence of the Holstein on the *arvalis*-group diversification can be explained by two historical scenarios. First, the preceding period, MIS 12, was characterized by a pronounced cold period (around 0.460 Mya), during which the earliest pan-Eurasian mammoth fauna associated with tundra-steppe habitats (called mammoth steppe, see Guthrie 2001) was formed. Second, the warmest phase of MIS 11 is the phase with the highest temperatures in the last 500 thousand years, persisting, persisting two times longer than the Eemian interglacial and three times longer than the Holocene (Sirocko et al 2007). Interglacial conditions may have disrupted the mammoth steppe biome due to an increase in precipitation, temperature, and associated forest expansions (for Late Quaternary see Řičánková et al. 2018). Tougard et al. (2008) recognized that the evolutionary history of temperate small mammals is much more complex than previously suggested. Individual species responded to various factors in multiple ways, and at different times during the Pleistocene (Lorenzen et al. 2011). Therefore, we tend to be reserved about whether observed pulses in diversification could be interpreted as expansion alongside some geographical/biotope barriers or fragmentation of some particular populations.

To conclude, our study proved an additional occurrence of *Microtusmystacinus* in Kazakhstan. The studies of Kovalskaya (1994), Meyer et al. (1996) and Okulova et al. (2014) specified the distribution of this species from western or northwestern parts of Kazakhstan, with the easternmost observation from the Karabalyk district (Kovalskaya 1994). Other localities of this species are known around Novosibirsk, several hundred kilometres away from the Kazakhstani border (Pavlova and Tchabovsky 2011). Although our material is not suitable to establish the full distribution range in Kazakhstan, it enables us to extend the range of this species further south.

The distribution of *Microtusmystacinus* could be partly human-induced, as documented by Tiunov et al. (2013) when regarding the railway across Siberia and the Far East of Russia (e.g., Olkhon Island, Pavlova and Tchabovsky 2011; Buryatia, Moroldoev et al. 2017). If we consider this possibility, the locality near Sekisovka is approx. 30 km distant from the nearest railway from Ust-Kamenogorsk to Ridder, but our second locality (near Dzhambul) is more than 150 km distant from the nearest railway at Zyryanovsk (built after 1930; according to official web page of KTZ – КАЗАКСТАН TEMIP ЖОЛЫ). In Russian territory, this species shows pathways of invasion around the Transbaikalia railway and the surrounding agricultural landscape (e.g., Tiunov et al. 2013, Moroldoev et al. 2017). As the Kazakhstani specimens are significantly divergent from other available sequences (approx. 100 kya), we could consider the distribution of *Microtusmystacinus* in Kazakhstan as natural, but additional evidence is welcomed. Based on the presented network-phylogenetic relationship of samples it seems that a potential route of colonization for Kazakhstan populations could have originated somewhere between the Balkans and sites north of the Black and Caspian seas, whereas populations in Turkey and parts of Armenia were colonized from a southern route.

Our study is the first genotyping of *Microtusmystacinus* from the eastern part of its distribution, where its’ occurrence is more discontinuous. In the context of our study, it is important to analyse genetically these Baikal and Far Eastern populations, and further map out the extent of *Microtusmystacinus* occurrence in East Kazakhstan.
